# A Divide-and-Conquer Embedded Correlated Wavefunction Transfer Learning Approach for Chemically Accurate Condensed-Phase Interatomic Potentials: Liquid Water as a Test Case

**DOI:** 10.1021/acs.jpclett.6c02897

**Published:** 2026-09-29

**Authors:** Xuezhi Bian, Emily A. Carter

**Affiliations:** † Department of Chemistry, 6740 Princeton University, Princeton, New Jersey 08544, United States; ‡ Department of Mechanical and Aerospace Engineering, Princeton University, Princeton, New Jersey 08544, United States; § Andlinger Center for Energy and the Environment and Program in Applied and Computational Mathematics, Princeton University, Princeton, New Jersey 08544, United States

## Abstract

Combining the accuracy of correlated wavefunction theory with the efficiency of machine-learned interatomic potentials offers a promising route toward predictive condensed-phase dynamics. In our previous work [ J. Chem. Theory Comput. 2026, 22­(10), 5174−5184, 10.1021/acs.jctc.6c00403 PMC1321754342095568], we introduced an embedded correlated wavefunction transfer learning (ECW-TL) approach, which efficiently refines a machine-learned potential to correlated-wavefunction accuracy within a predefined region containing the chemistry of interest. Although effective for localized processes such as ion pairing in solution, this formulation is not directly applicable to systems whose behavior and properties emerge collectively and cannot be confined to a selected region. Here, we overcome this limitation by developing a divide-and-conquer formulation of ECW-TL that partitions the system into embedded subsystems, enabling correlated-wavefunction corrections to be learned throughout the entire simulation cell. We demonstrate the method using ambient liquid water as a test case because of its diverse, collectively fluctuating hydrogen-bonding environments. The resulting potential fine-tuned at the embedded-CCSD­(T) level improves the structural, thermodynamic, and dynamical properties of liquid water relative to underlying DFT models, bringing calculated observables closer to experiment. These results suggest that divide-and-conquer ECW-TL provides a promising, scalable route toward accurate simulations of complex condensed-phase systems.

## Graphical abstract

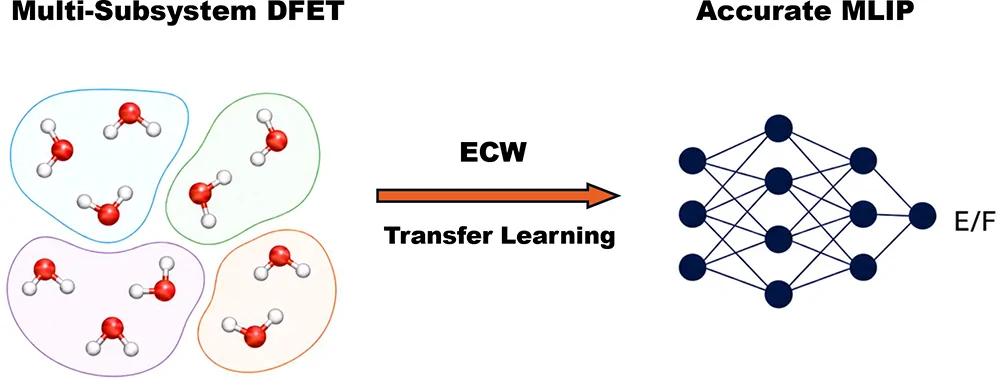

Machine-learned interatomic potentials (MLIPs) have made large-scale, first-principles quality condensed-phase molecular dynamics (MD) simulations increasingly accessible.^1–3^ Their reliability, however, ultimately is limited by the electronic structure theory used to generate the training data. Most MLIP-based simulations rely on density functional theory (DFT) and therefore inherit the limitations of the underlying approximate exchange-correlation functional.^4,5^ In particular, electron self-interaction and delocalization errors can distort the potential-energy landscape and the resulting forces.^6–8^ These errors will propagate through MD trajectories, potentially biasing predicted properties.^9,10^ This limitation has motivated growing efforts to construct or refine MLIPs using more accurate electronic-structure methods.

Correlated wavefunction (CW) methods offer a natural route to improving MLIP accuracy by including exact exchange and electron correlation beyond approximate DFT.^11^ For example, coupled-cluster theory with single, double, and perturbative triple excitations [CCSD­(T)] has demonstrated benchmark accuracy for closed-shell, main-group chemistry.^12,13^ Extending CW accuracy to condensed-phase MLIPs, however, remains prohibitively demanding. Typical CW calculations generally scale steeply with system size,^14^ and the analytical energy gradients required for force training are computationally expensive or unavailable for many reduced-scaling CW methods^15–18^ applicable to larger systems. Consequently, direct training on CW data has largely been restricted to small molecular data sets and gas-phase dynamics,^19–21^ with comparatively few applications to extended systems.^22^

Despite these challenges, considerable progress has been made recently toward constructing CW-accurate MLIPs through data-efficient Δ-learning^23–25^ and transfer-learning approaches.^26–29^ The central idea is to retain the potential-energy landscape learned from a lower-level reference, typically a DFT-based MLIP, and learn only the correction from DFT to the target CW method. This correction is generally smoother and more data-efficient to represent than the full high-level potential. Nevertheless, generating CW reference data for complete periodic cells remains computationally challenging. Existing approaches are therefore often restricted to small periodic training cells^30^ or rely on gas-phase clusters extracted from the condensed-phase system.^31,32^ These isolated cluster calculations neglect interactions with the surrounding chemical environment and require increasingly large clusters to approach convergence.^33,34^ Moreover, MLIPs trained on such data may encounter configurations during condensed-phase MD that are poorly represented by the isolated clusters, resulting in unstable trajectories or inaccurate predictions of properties such as equilibrium density.^35,36^

In our recent work,^37^ we introduced an embedded correlated wavefunction transfer learning (ECW-TL) framework to address these limitations. ECW-TL employs density functional embedding theory (DFET) to represent the interaction between a selected cluster and its surrounding environment through a physically grounded embedding potential. CW calculations are then performed on the embedded cluster, retaining a quantum-mechanical description of its coupling to the condensed phase, and the resulting higher-level quantum data are used to refine the MLIP. Related ideas have also emerged in range-separated learning approaches based on semiempirical quantum mechanics/molecular mechanics (QM/MM) embedding.^38^ We demonstrated ECW-TL for calcium–carbonate ion pairing, where it successfully reproduced target-level structures and free-energy profiles in controlled DFT-to-DFT transfer-learning tests. Extension to CW reference data further yielded ion-pairing thermodynamics in good agreement with experiment, demonstrating the framework’s ability to translate high-level electronic-structure accuracy into reliable condensed-phase dynamics. That being said, the original ECW-TL framework applies the higher-level correction only within a predefined region containing the chemistry of interest. Although well suited to localized processes such as ion pairing in solution, this formulation assumes that the relevant higher-level effects can be confined to a selected region. The choice and size of this region are therefore not unique and must be tested for convergence. More importantly, the local formulation is not readily applicable when relevant molecular environments evolve throughout the system or when the properties of interest arise from collective, nonlocal behavior. These limitations motivate a framework that can distribute embedded CW information throughout the simulation cell and thereby refine the potential-energy surface globally.

Here, we extend ECW-TL beyond a predefined region of interest through a divide-and-conquer approach based on multisubsystem DFET. The periodic simulation cell is partitioned into multiple subsystems that interact through a common, globally determined embedding potential. The embedded subsystems then can be treated independently at the target higher level of theory and their CW corrections combined to refine the MLIP for the entire simulation cell. This construction enables high-level ECW corrections to be incorporated systematically into systems governed by collective dynamics and spatially nonlocal correlations. We demonstrate the framework using liquid water at ambient conditions. As the primary medium for a vast range of chemical and biological processes, liquid water is both fundamentally important and exceptionally well characterized experimentally. At the same time, its properties emerge from a cooperative, spatially extended, and continuously fluctuating hydrogen-bond network, making it a perfect test of the present approach. We first introduce the methodology and establish its accuracy through controlled DFT-to-DFT tests, for which full-system reference calculations are available. We then apply the framework at the CW level and evaluate the resulting potential against experimental structural, thermodynamic, and dynamical properties. These results demonstrate the potential of divide-and-conquer ECW-TL as a scalable route toward chemically accurate condensed-phase molecular dynamics simulations.

## Multisubsystem DFET/ECW Theory

Quantum embedding methods provide a powerful route to high-accuracy electronic structure calculations at reduced computational cost by treating a chemically relevant region at a high level of theory while retaining the influence of its surrounding environment.^39,40^ Among many approaches, density functional embedding theory^41–43^ (DFET) and embedded correlated wavefunction^44^ (ECW) theory offer a practical framework for extending CW methods to complex extended systems. These methods have been successfully applied to diverse phenomena, including plasmonic excited-state chemistry,^45–50^ electrocatalytic reactions at metal surfaces,^51–56^ and reactions in aqueous solutions.^57–61^

In DFET, the full system is partitioned into interacting subsystems, typically two: a cluster and its environment that are coupled through a unique embedding potential, as illustrated in Figure 1(a). This potential is optimized such that the sum of the subsystem densities reproduces the full-system reference density. Mathematically, this optimized effective potential (OEP) method can be expressed as maximization of an extended Wu-Yang functional^62^ *W* with respect to the embedding potential *V*_emb_:

1 $$\begin{array}{l} W\left[V_{\mathrm{emb}}\right]=E_{\mathrm{cluster}}^{\mathrm{DFT}}\left[\rho_{\mathrm{cluster}},V_{\mathrm{emb}}\right]+E_{\mathrm{enviro}}^{\mathrm{DFT}}\left[\rho_{\mathrm{enviro}},V_{\mathrm{emb}}\right]-\int \;dr\;V_{\mathrm{emb}}\left(r\right)\rho_{\mathrm{tot}}\left(r\right) \end{array}$$

where *E*_cluster_^DFT^ and *E*_enviro_^DFT^ are self-consistent DFT energies of the cluster and its environment subject to *V*_emb_, ρ_cluster_ and ρ_enviro_ represent the corresponding electron densities, and ρ_tot_ is the reference density of the total system. At convergence, the sum of electron density distribution of the cluster and environment is equal to the entire periodic cell reference density distribution:

2 $$\rho_{\mathrm{tot}}\left(r\right)=\rho_{\mathrm{cluster}}\left[V_{\mathrm{emb}}\right]\left(r\right)+\rho_{\mathrm{enviro}}\left[V_{\mathrm{emb}}\right]\left(r\right)$$

**Figure 1:**
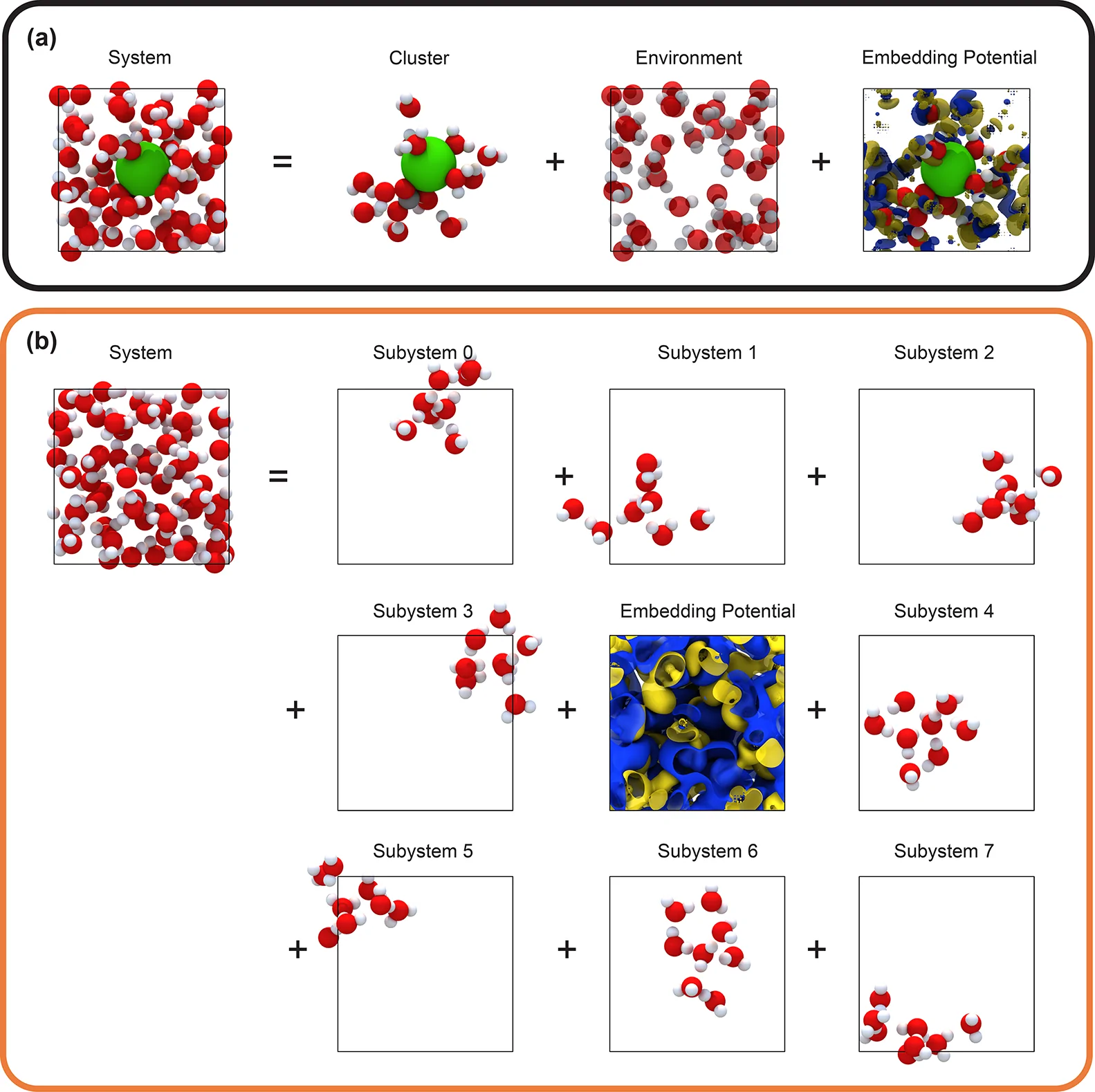
Schematic illustration of (a) two-component cluster/environment DFET and (b) multi-subsystem DFET. In (a), the structure is taken from our previous study,^37^ with the cluster defined as the solutes plus the first solvation shell of the calcium–carbonate ion pair (Ca depicted as green sphere, O red, H white, C gray). The embedding potential, shown as blue and yellow isosurfaces at ± 0.3 V, is localized around the cluster and represents its interaction with its extended environment. In (b), a periodic cell containing 64 water molecules is partitioned into eight subsystems of eight molecules each. For visualization purposes, each subsystem is unwrapped across the periodic boundaries so that its water molecules are displayed as a spatially contiguous cluster. At the same isosurface values, the embedding potential is distributed throughout the cell, reflecting the interaction of each subsystem with all other subsystems.

This density-matching condition ensures that the interaction between the cluster and its environment is represented exactly at the DFT level. Then, in ECW theory, the embedding potential is incorporated into the ECW Hamiltonian of the cluster as an additional one-electron operator:

3 $${Ĥ}_{\mathrm{cluster}}^{\mathrm{ECW}}={Ĥ}_{\mathrm{cluster}}^{\mathrm{CW}}+{\mathrm{V̂}}_{\mathrm{emb}}$$

where *Ĥ*_cluster_^CW^ is the isolated cluster’s correlated wavefunction Hamiltonian. The resulting ECW calculation therefore combines a correlated wavefunction description of the cluster with the quantum-mechanical environmental effects encoded in the embedding potential, and the corresponding energy correction in ECW theory is defined as

4 $$E_{\mathrm{system}}^{\mathrm{ECW}}=E_{\mathrm{system}}^{\mathrm{DFT}}+\left(E_{\mathrm{cluster}}^{\mathrm{ECW}}-E_{\mathrm{cluster}}^{\mathrm{emb}-\mathrm{DFT}}\right)$$

Here *E*_system_^DFT^ is the total energy of the periodic system calculated at DFT level; *E*_cluster_^ECW^ is obtained by solving the ECW Hamiltonian in eq 3; and *E*_cluster_^emb‑DFT^ is the DFT energy of the embedded cluster. The ECW and embedded DFT calculations are typically performed using the same atomic-orbital basis set and effective core potentials or pseudopotentials. This consistent treatment provides a well-defined ECW formalism for incorporating CW-level energy corrections into the full-system DFT energy.

Above, we briefly reviewed the basic formulation of DFET/ECW theory. However, although most previous ECW calculations have employed a two-subsystem partitioning including a target cluster and its environment, this choice is not a fundamental constraint of the theory. More generally, DFET allows the full system to be partitioned into an arbitrary number of interacting fragments or subsystems, all coupled through a single common and unique embedding potential, as illustrated in Figure 1(b). We note that the uniqueness proof in ref 41 based on two subsystems can be directly extended to an arbitrary number of them. The OEP procedure then can be obtained by maximizing a multi-subsystem extended Wu-Yang functional:

5 $$W\left[V_{\mathrm{emb}}\right]=\underset{i}{\sum }E_{\mathrm{subsystem},i}^{\mathrm{DFT}}\left[\rho_{\mathrm{subsystem},i},V_{\mathrm{emb}}\right]-\int dr\;V_{\mathrm{emb}}\left(r\right)\rho_{\mathrm{tot}}\left(r\right)$$

where *E*_subsystem,*i*_^DFT^ and ρ_subsystem,*i*_ are the self-consistent DFT energy and electron density of the *i*-th subsystem in the presence of the embedding potential, respectively. At convergence, the sum of the subsystem densities reproduces the full-system reference density:

6 $$\rho_{\mathrm{tot}}\left(r\right)=\underset{i}{\sum }\rho_{\mathrm{subsystem},i}\left[V_{\mathrm{emb}}\right]\left(r\right)$$

For each embedded subsystem, the optimized embedding potential then can be incorporated into its CW Hamiltonian to perform an ECW calculation separately:

7 $${Ĥ}_{\mathrm{subsystem},i}^{\mathrm{ECW}}={Ĥ}_{\mathrm{subsystem},i}^{\mathrm{CW}}+{\mathrm{V̂}}_{\mathrm{emb}}$$

The ECW energy corrections from all embedded subsystems can be combined to correct the full-system DFT energy following the same spirit as in eq 4:

8 $$E_{\mathrm{system}}^{\mathrm{ECW}}=E_{\mathrm{system}}^{\mathrm{DFT}}+\underset{i}{\sum }\left(E_{\mathrm{subsystem},i}^{\mathrm{ECW}}-E_{\mathrm{subsystem},i}^{\mathrm{emb}-\mathrm{DFT}}\right)$$

Together, eqs 5–8 constitute the working equations of the multi-subsystem extension of DFET/ECW theory. Unlike the conventional cluster/environment formulation, this extension does not assume that high-level corrections can be confined to a predefined chemically important region. Instead, ECW corrections are distributed across the entire simulation cell through its constituent subsystems, systematically refining the full-system energy toward CW-level accuracy. This divide-and-conquer strategy retains the influence of the extended chemical environment while avoiding the steep computational scaling of direct periodic CW calculations on the full system. It therefore provides a computationally feasible framework for extending CW accuracy to intermediate-sized, condensed-phase systems and, importantly, for generating consistent high-accuracy reference energies for training MLIPs.

## Divide-and-Conquer ECW-TL

To construct a practical, high-accuracy MLIP, we extend the ECW-TL framework established in our previous work to the multi-subsystem DFET/ECW formulation. As discussed above, current ECW calculations provide energies but not forces, and their computational cost limits the number of configurations for which reference ECW energies can be computed and used as training data (in machine-learning parlance, “labelled”). A data-efficient learning strategy is therefore essential for translating these sparse, energy-only ECW labels into an accurate MLIP. The resulting divide-and-conquer ECW-TL workflow retains the same five general stages:

(1) **Baseline DFT-MLIP training**. A baseline DFT-MLIP is trained until it accurately describes the DFT potential-energy surface across the configuration space relevant to the system and target properties.
(2) **Representative configurations selection**. A compact and structurally diverse set of configurations is selected for the more expensive ECW calculations. These configurations may be selected from the converged DFT training data set, MD trajectories generated using the baseline DFT-MLIP, or both. The selection could be guided by chemical intuition and structural descriptors that characterize the sampled atomic environments, as in our previous work,^37^ or by committee-model deviations, as in the present work.
(3) **Multi-subsystem ECW labeling.** Each selected configuration is partitioned into a set of subsystems that collectively span the full simulation cell. This partitioning requires careful consideration of the subsystem size, chemical composition, and boundary placement, with the goal of maintaining chemically meaningful and consistent subsystem definitions across the training configurations. Further details of this work are discussed in the results section below. Once the subsystems are defined, a multi-subsystem DFET calculation is performed to determine the common embedding potential, followed by embedded DFT and ECW calculations for each subsystem. The resulting subsystem corrections are combined according to eq 8 to construct the ECW-corrected energy of the full configuration.
(4) **Transfer learning.** The baseline DFT-MLIP is fine-tuned using the multi-subsystem ECW energy labels. Here and throughout this work, “fine-tuning” refers exclusively to transfer learning with higher-level electronic-structure data; no experimental observables are used in model training. The fine-tuning procedure and hyperparameters should be carefully chosen to balance learning the ECW-level corrections with retaining the configurational knowledge acquired from the larger DFT data set.
(5) **Validation and iterative refinement.** The divide-and-conquer ECW-TL model is evaluated using target properties of interest. If the required accuracy or convergence has not been achieved, additional configurations are selected and labeled through multi-subsystem DFET/ECW calculations. The transfer-learning and validation steps are then repeated until the model and target properties are sufficiently converged.

Overall, this workflow removes the restriction of the original ECW-TL approach to localized chemical processes and provides a practical route to MLIPs informed by ECW corrections across an extended system. It therefore enables high-accuracy simulations of nonlocal collective behaviors in condensed-phase dynamics.

We use liquid water at ambient conditions as the first application of the divide-and-conquer ECW-TL framework. As the primary medium for biological, chemical, and environmental processes, water is both scientifically important and exceptionally well characterized. It also exhibits remarkable properties, including a density maximum near the melting point, lower-density ice, a large static dielectric constant, and anomalous transport behavior.^63^ These properties reflect the cooperative and continuously fluctuating hydrogen-bond network, whose collective structural rearrangements and molecular reorientations are further influenced by nuclear quantum effects.^64^

First-principles descriptions of liquid water have advanced substantially,^65^ progressing from DFT-based first-principles molecular dynamics^10,66,67^ to many-body potentials^68,69^ and, more recently, MLIPs.^70,71^ These developments have greatly expanded the accessible length and time scales, yet important discrepancies with experiment remain. Many commonly used DFT approximations predict an overly structured hydrogen-bond network and excessively slow diffusion, while properties such as the equilibrium density,^72^ melting temperature,^73^ dielectric response,^74^ and relative stability of liquid water and ice remain strongly dependent on the exchange-correlation functional.^75^ MLIPs enable these properties to be evaluated with much better statistical convergence, but models trained on DFT data inherit the same functional-dependent biases. Recent MLIPs trained on CW data have substantially improved several predictions,^31,34^ but simultaneously reproducing water’s structural, thermodynamic, dielectric, and dynamical properties remains challenging. Liquid water therefore provides an ideal test of whether divide-and-conquer ECW-TL can translate CW-level energetics into improved predictions of condensed-phase properties at a much lower cost.

## Validation

Before applying the framework at the ECW level, we first validate it through a controlled DFT-to-DFT test. We construct two DFT-based MLIPs using the Deep Potential^76,77^ (DP) model implemented in DeepMD-kit.^78^ One is trained using the revised Perdew–Burke–Ernzerhof^79,80^ exchange-correlation functional with the D3 dispersion correction^81,82^ and Becke–Johnson damping^83^ [revPBE-D3­(BJ)], and the other using the strongly constrained and appropriately normed^84^ (SCAN) functional. All periodic plane-wave DFT calculations are performed using VASP.^85–87^ Both models are iteratively refined using the active-learning workflow implemented in DP-GEN,^88^ which consists of model training, configurational exploration, and DFT labeling. During exploration, a committee of independently trained MLIPs estimates model uncertainty from the deviations among their force predictions. Configurations exceeding the prescribed force-deviation threshold are selected for DFT labeling and added to the training set. This cycle is repeated until the sampled configurations exhibit sufficiently low model uncertainty. Computational and training details for all DFT and MLIP models are provided in the Supporting Information (SI).

In this validation test, the generalized-gradient-approximation (GGA)-level DFT-revPBE-D3­(BJ) model serves as the baseline, whereas the meta-GGA DFT-SCAN model provides the higher-level reference. The goal is to refine the baseline model toward SCAN accuracy using the divide-and-conquer ECW-TL framework. After active learning is converged over the relevant temperature and pressure ranges for classical MD, the DFT-revPBE-D3­(BJ) training data set gathers 665 configurations contain 64 waters each. As an initial validation, all configurations in this data set are subjected to multi-subsystem DFET calculations, with embedded SCAN corrections providing the energy labels for transfer learning.

Although the multi-subsystem DFET/ECW theory incorporates high-level corrections across the full periodic cell, the resulting ECW energy correction remains dependent on how the system is partitioned. This is because interactions within each subsystem are corrected at the CW level, but interactions between different subsystems remain described through the DFT-level embedding potential. Therefore, the partition should be constructed so that the dominant short-range interactions are contained within individual subsystems. This consideration is particularly important for liquid water, where molecular diffusion and continuous rearrangement of the hydrogen-bond network preclude a fixed partition based on molecular identities. We thus construct spatially local subsystems independently for each configuration while keeping their sizes consistent to ensure a consistent level of embedding.

Here and throughout this work, we apply the high-level correction to a periodic cell containing 64 water molecules, partitioned into eight subsystems of eight water molecules each. The partition is generated geometrically: eight central water molecules are first selected by maximizing their mutual separation. The remaining water molecules are then assigned to these centers by minimizing the average distance to their respective central molecules with the constraint that each subsystem contains exactly eight water molecules. This procedure generates compact, uniformly sized subsystems and retains the dominant local intermolecular interactions within individual subsystems, where they are treated at the CW level. Although some ambiguity remains in the precise partitioning, in practice we find that its influence on the calculated energy correction is negligible for liquid water, provided that the subsystems are constructed to remain spatially local.

The choice of eight subsystems containing eight water molecules each balances accuracy and computational cost. Larger subsystems include more intermolecular interactions at the CW level and are therefore expected to improve accuracy, but their computational cost increases much faster than linearly with subsystem size in CW theory. The eight-molecule subsystem provides a practical compromise, yielding acceptable accuracy while keeping high-level CW calculations, such as CCSD­(T), computationally tractable.

Once the partition is defined, a multi-subsystem DFET calculation is performed for each configuration. A common embedding potential is optimized such that the sum of the subsystem electron densities reproduces the full-cell DFT-revPBE-D3­(BJ) density. Under this optimized embedding potential, each subsystem is evaluated at both the baseline DFT-revPBE-D3­(BJ) and target DFT-SCAN levels. The resulting subsystem energy differences are summed and combined with the full-cell baseline energy according to eq 8 to obtain the embedded DFT-SCAN-corrected energy of the full system. The DFET and embedded plane-wave DFT calculations are performed using a modified version of VASP,^89^ with further computational details provided in the SI.

For comparison, the baseline and target energies of the isolated subsystems are also evaluated without the embedding potential. Their energy differences are then summed and combined with the full-cell baseline energy in the same manner, yielding an unembedded “ONIOM-type” correction:^90^

9 $$E_{\mathrm{system}}^{\mathrm{ONIOM}}=E_{\mathrm{system}}^{\mathrm{revPBE}-D3\left(\mathrm{BJ}\right)}+\underset{i}{\sum }\left(E_{\mathrm{subsystem},i}^{\mathrm{SCAN}}-E_{\mathrm{subsystem},i}^{\mathrm{revPBE}-D3\left(\mathrm{BJ}\right)}\right)$$

The two corrected data sets, embedded-SCAN and ONIOM-SCAN, are then used separately to fine-tune the baseline DFT-revPBE-D3­(BJ) model.

We find that energy-only transfer learning is particularly sensitive to the batch size. A relatively large batch size, e.g., 100 configurations, is required to obtain accurate results. In conventional energy-and-force training, each configuration provides one energy and 3*N*_atom_ force labels, whereas energy-only training provides only one scalar label per configuration. A larger batch therefore supplies a more statistically informative and less noisy gradient for each stochastic optimization step. We also use a small initial learning rate of (10^–6^) in transfer learning, as excessively large parameter updates can destabilize the pretrained model and cause catastrophic forgetting of the baseline potential-energy surface. For liquid water in this study, the transfer-learning results are less sensitive to other hyperparameters or training procedures, such as freezing selected network layers or varying the total number of training steps. Further details of the transfer-learning procedure, including systematic tests of batch size and subsystem size, are provided in the SI. The results show that accuracy improves with increasing subsystem size and is nearly converged for subsystems containing eight water molecules.

Using the two DFT-based models and the two fine-tuned models, we perform classical molecular dynamics simulations of liquid water at 298 K in the canonical (NVT) ensemble using LAMMPS.^91^ The cubic simulation cell contains 512 water molecules at the experimental density of 0.997 g/cm^3^. Each simulation is performed for 2 ns using the Nosé-Hoover chain thermostat^92^ with a damping time of 0.1 ps. Unless otherwise specified, a time step of 0.5 fs is used for all MD simulations throughout this work. The resulting trajectories are used to compare the structural and dynamical properties predicted by DFT-revPBE-D3­(BJ), DFT-SCAN, embedded-SCAN, and ONIOM-SCAN, thereby assessing how accurately the two transfer-learning approaches reproduce the target DFT-SCAN model.

We examine the oxygen–oxygen radial distribution function (RDF) *g*_*OO*_ (*r*), which characterizes the intermolecular structure of liquid water. As shown in Figure 2, the DFT-SCAN model predicts a substantially more structured liquid than the baseline DFT-revPBE-D3­(BJ) model, with more pronounced first and second peaks. The embedded-SCAN fine-tuned model quantitatively reproduces the DFT-SCAN RDF, with the two curves lying nearly on top of each other, thereby validating the divide-and-conquer ECW-TL framework. In contrast, the ONIOM-SCAN fine-tuned model shifts the RDF from the baseline prediction toward the DFT-SCAN result but remains intermediate between the two. The superior performance of embedded-SCAN highlights the importance of the embedding potential for accurately capturing the influence of the surrounding environment on each subsystem.

**Figure 2:**
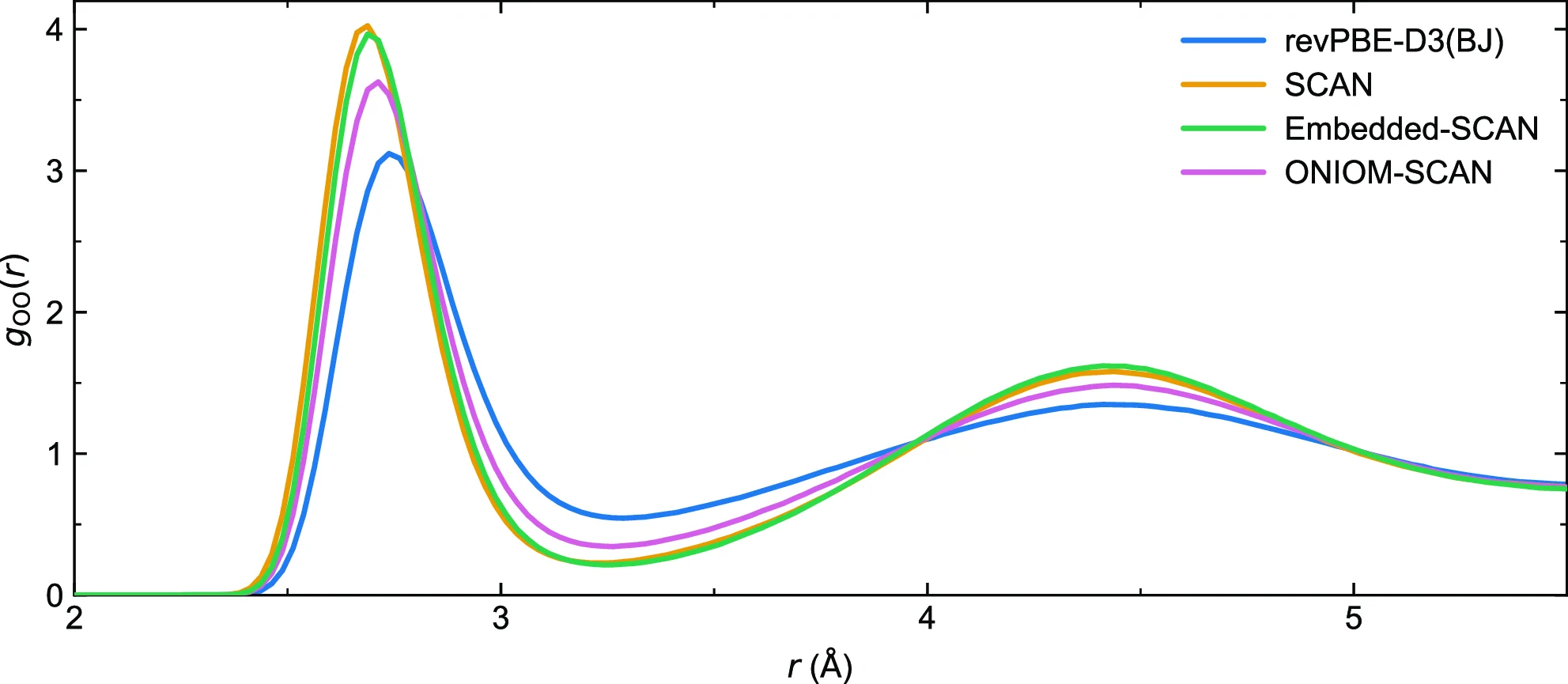
Oxygen–oxygen radial distribution functions *g*_*OO*_ (*r*) of liquid water obtained from classical NVT-MD simulations at 298 K and the experimental density using the DFT-revPBE-D3­(BJ), DFT-SCAN, embedded-SCAN, and ONIOM-SCAN models. The embedded-SCAN model quantitatively reproduces the target DFT-SCAN result, whereas ONIOM-SCAN predicts an intermediate structure between the baseline DFT-revPBE-D3­(BJ) and target DFT-SCAN models.

## Application

Following the successful DFT-to-DFT validation, we next apply the divide-and-conquer ECW-TL framework at the CW level. To obtain a baseline model suitable for physical simulations, we further refine the DFT-revPBE-D3­(BJ) model through additional active-learning cycles that include configurations sampled by path-integral molecular dynamics (PIMD), thereby expanding the training domain to account for nuclear quantum fluctuations. The resulting baseline data set contains 2,379 configurations, making CW calculations for the entire data set computationally prohibitive. We therefore select a diverse subset of configurations with large force deviations, as predicted by a committee of four independently trained models from the last active learning iteration. The candidate pool includes both the existing training configurations and configurations sampled from MD trajectories at 300 and 350 K with the baseline DFT-revPBE-D3­(BJ) model. Additional model energy and force validation and controlled DFT-to-DFT tests from DFT-revPBE-D3­(BJ) to DFT-SCAN, presented in the SI, indicate that 300 configurations are sufficient for accurate energy-only transfer learning. Based on this validation, we use the same number of configurations for transfer learning at the CW level.

Multi-subsystem DFET calculations at DFT-revPBE-D3­(BJ) level are then performed for the selected configurations following the partitioning scheme described in the validation section. After each embedding potential is obtained in the periodic plane-wave DFT, it is projected onto a Gaussian-type-orbital (GTO) basis for the subsequent ECW calculations. Because the spatially local subsystems are sufficiently separated from their periodic images, each embedded subsystem can be treated as an isolated cluster. This differs from our previous work, in which the larger subsystems require periodic GTO calculations. For each subsystem, embedded CCSD­(T) and DFT-revPBE-D3­(BJ) calculations are performed using the cc-pVTZ basis set,^93^ with all GTO-based electronic structure calculations carried out in PySCF.^94^ Based on previous CCSD­(T) studies of water clusters, the triple-ζ basis is expected to capture the dominant correlation contribution.^95^ The 300 selected configurations, each partitioned into eight subsystems, require a total of 2,400 isolated eight-water CCSD­(T) calculations, which remains computationally tractable. Using the 300 embedded CCSD­(T)-corrected energy labels and the fine-tuning procedure established in the validation study, we obtain the final production ECW-TL-CCSD­(T) model.

To assess the quality of the models, we perform PIMD simulations in the NVT ensemble at 298 K using the DFT-revPBE-D3­(BJ), DFT-SCAN, and ECW-TL-CCSD­(T) models. The simulations employ the same cubic cell containing 512 water molecules at a density of 0.997 g/cm^3^. Each PIMD trajectory is propagated for 2 ns using 32 ring-polymer beads to account for the nuclear quantum effect and a Langevin thermostat^96,97^ with a relaxation time of 0.1 ps. We first look at the structural properties of liquid water through the oxygen–oxygen, oxygen–hydrogen, and hydrogen–hydrogen radial distribution functions, (*g*_*OO*_ (*r*), *g*_*OH*_ (*r*) and *g*_*HH*_ (*r*)), respectively, and compare them directly with experimental measurements. The results are shown in Figure 3.

**Figure 3:**
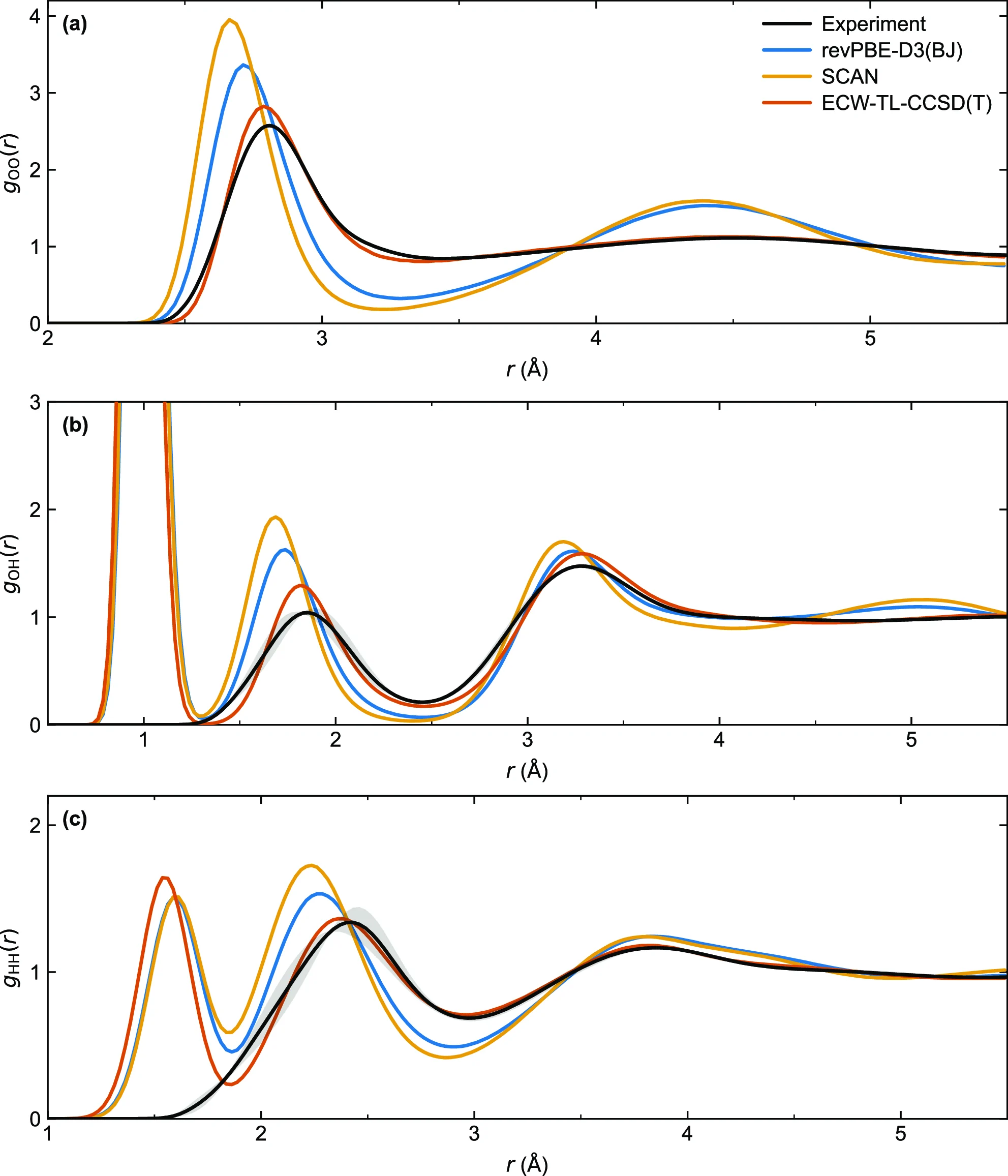
(a) Oxygen–oxygen RDFs *g*_*OO*_ (*r*) of liquid water obtained from NVT-PIMD simulations at 298 K and the experimental density using the DFT-revPBE-D3­(BJ), DFT-SCAN, ECW-TL-CCSD­(T) models, compared with the experimental result from ref 98. The shaded region indicates experimental uncertainty. (b, c) Corresponding oxygen–hydrogen and hydrogen–hydrogen RDFs, (*g*_*OH*_ (*r*) and *g*_*HH*_ (*r*)), compared with the experimental results from ref 99. For all three RDFs, ECW-TL-CCSD­(T) agrees more closely with experiment than the DFT-based MLIP models. The reference O–H and H–H RDFs, derived from neutron and X-ray scattering experiments,^99^ include only intermolecular contributions and omit intramolecular peaks.

As shown by *g*_*OO*_ (*r*) in Figure 3(a), both DFT-based models predict a substantially overstructured liquid relative to experiment, with overly pronounced first- and second-coordination shell peaks. These limitations of semilocal DFT descriptions of liquid water have been extensively reported in previous studies.^10,100,101^ In particular, SCAN overstructuring has been associated with density-driven errors arising from self-interaction and electron delocalization, which lead to overbinding of the dominant two-body water interactions.^102^ For revPBE-D3, previous studies have shown that its description of water depends on error cancellation between density-driven and functional-driven contributions and is sensitive to the chosen dispersion correction.^103^ Similar trends are observed for *g*_*OH*_ (*r*) and *g*_*HH*_ (*r*) in Figure 3(b) and (c). Both DFT-based models overestimate the intermolecular hydrogen-bond peaks relative to experiment, indicating excessively strong O···H positional correlations and orientational ordering between neighboring water molecules. In contrast, by providing a more accurate description of the local intermolecular interactions, the ECW-TL-CCSD­(T) model substantially reduces the excessive ordering and yields consistently improved agreement with experiment for all three site–site RDFs.

With the improved structural description provided by the ECW-TL-CCSD­(T) model, we next turn to dynamical and thermodynamic properties by examining the self-diffusion coefficient and equilibrium density of liquid water at different temperatures. Classical MD simulations are performed in the isothermal–isobaric (NPT) ensemble at 1 bar over the temperature range of 280–310 K. Pressure is controlled using a Martyna-Tobias-Klein^104^ barostat with a relaxation time of 0.5 ps, and each trajectory is propagated for 10 ns. The self-diffusion coefficient *D* is determined from the long-time slope of the oxygen mean-squared displacement (MSD) using the Einstein relation,

10 $$D=\underset{t\to \infty }{\lim}\frac{1}{6}\frac{d}{dt}{\left|r\left(t\right)-r\left(0\right)\right|}^{2}$$

with the slope obtained by linear fitting over the time window from 0.5 to 2.0 ns. The results are shown in Figure 4. Both DFT-based models significantly underestimate the diffusion constant in the investigated temperature range, with DFT-SCAN predicting values nearly an order of magnitude smaller than experiment. This pronounced dynamical slowdown is consistent with the overly stable and ordered hydrogen-bond networks evidenced by the RDFs above and with their previously reported elevated melting temperatures. In contrast, ECW-TL-CCSD­(T) captures both the correct order of magnitude and the experimental temperature dependence of the self-diffusion coefficient.

**Figure 4:**
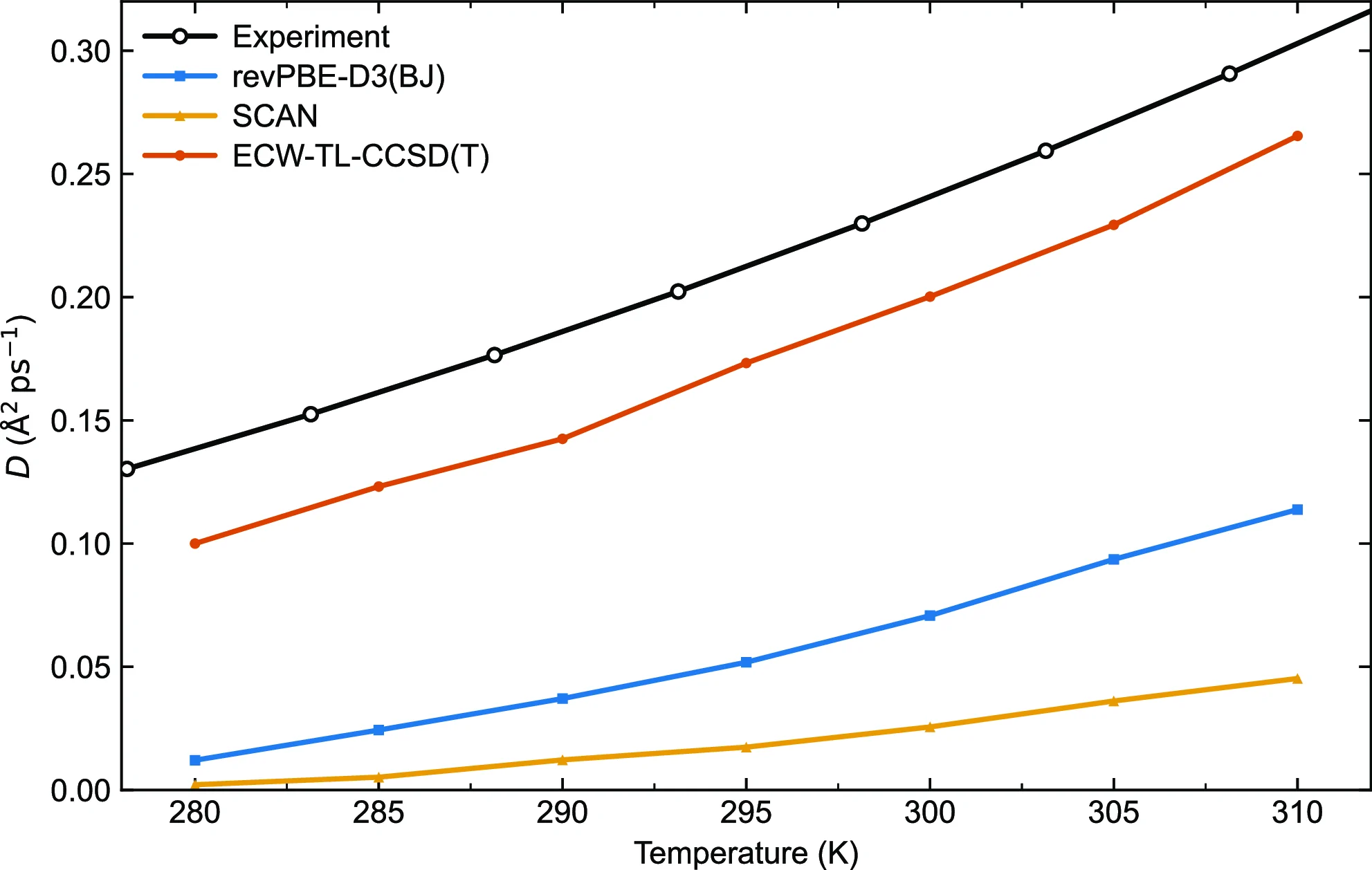
Temperature-dependent self-diffusion coefficients of liquid water calculated from classical NPT-MD simulations using the DFT-revPBE-D3­(BJ), DFT-SCAN, and ECW-TL-CCSD­(T) models, compared with experimental data from ref 105. Although all three models underestimate the experimental values, ECW-TL-CCSD­(T) provides improved agreement across the investigated temperature range.

Using the same 10 ns classical NPT trajectories, we calculate the temperature-dependent equilibrium densities predicted by the three models, as shown in Figure 5. To assess the influence of nuclear quantum effects (NQEs), we additionally perform 2 ns NPT-PIMD simulations using the ECW-TL-CCSD­(T) model, with pressure controlled by a Bussi-Zykova-Parrinello barostat.^97,106^ We find that the DFT-revPBE-D3­(BJ) model underestimates the experimental density, whereas DFT-SCAN overestimates it in the investigated temperature range. Transfer learning with embedded CCSD­(T) corrections onto the DFT-revPBE-D3­(BJ) baseline shifts the predicted density toward experiment, and including NQEs further improves the agreement. We note that there is no CCSD­(T)-level virial or stress information included in the transfer learning procedure. The improved density is therefore learned entirely from energy corrections, with their volume dependence encoded indirectly by training configurations. This result is particularly encouraging because cluster-to-bulk transfer based on isolated clusters can produce substantial errors in equilibrium density. A recent cluster-based Δ-learning study required a cluster of more than 20 water molecules, to converge the density and other bulk properties.^34^ In contrast, the present approach uses embedded subsystems containing only eight water molecules, with the embedding potential retaining the influence of the surrounding condensed-phase environment. The density predictions could be improved further by explicitly including more training configurations spanning a broader range of volumes and densities.

**Figure 5:**
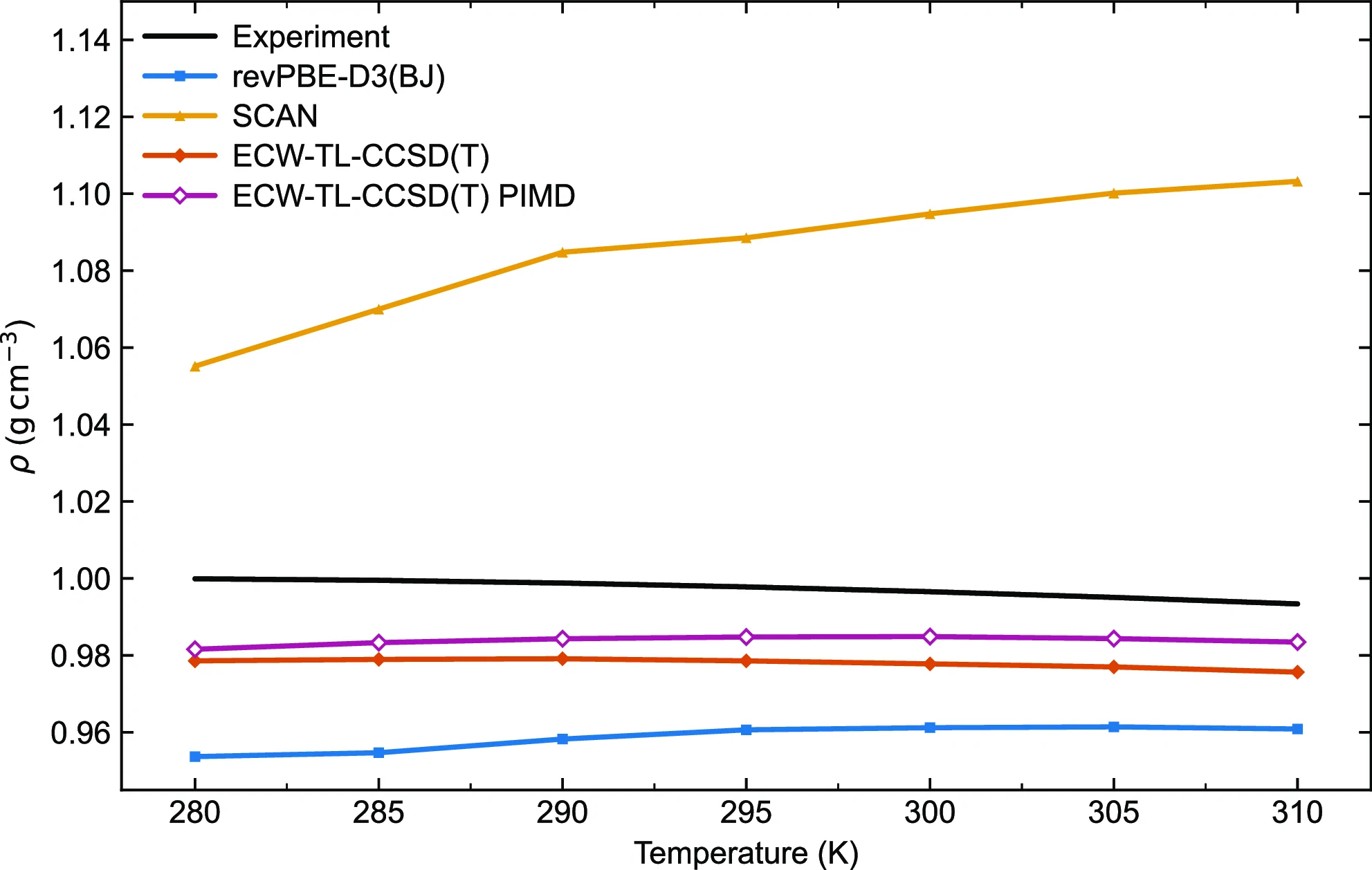
Temperature-dependent density of liquid water calculated from classical NPT-MD simulations using all three models and from quantum NPT-PIMD simulations using the ECW-TL-CCSD­(T) model, compared with experimental data from ref 107. DFT-revPBE-D3­(BJ) underestimates the density, whereas DFT-SCAN overestimates it. The ECW-TL-CCSD­(T) model improves the predicted density, with further improvement upon inclusion of nuclear quantum effects.

We next turn to the dielectric properties of liquid water, whose accurate calculation is particularly challenging for electronic structure methods and statistical convergence of MD simulations. In addition to the machine-learned energy and force models used for the MD simulations, we train two Deep Wannier^108^ (DW) models using maximally localized Wannier functions^109^ (MLWFs) obtained from DFT wavefunctions at DFT-revPBE-D3­(BJ) and DFT-SCAN level with Wannier90.^110^ The DW models predict the displacements of the electronic-charge centroids associated with each atom from its local chemical environment, enabling efficient calculation of the instantaneous total dipole moment along MD trajectories. Further details of the Wannierization procedure and DW-model training are provided in the SI. Using the same NPT trajectories employed in Figures 4 and 5, we first calculate the static dielectric constant *ε* from fluctuations of the total cell dipole moment:

11 $$\varepsilon =1+\frac{{\left\langle \left|M\right|\right\rangle }^{2}-{\left|\left\langle M\right\rangle \right|}^{2}}{3\varepsilon_{0}k_{B}T\left\langle V\right\rangle }$$

where ***M*** is the instantaneous total dipole moment calculated from the ionic and the Wannier centroid positions predicted by the DW model, *V* is the cell volume, and *ε*_0_ is the vacuum permittivity. For trajectories generated using DFT-based models, the corresponding DW model is used to calculate the dielectric response. The ECW-TL-CCSD­(T) results are calculated with the DFT-revPBE-D3­(BJ) DW model applied to the ECW-TL-CCSD­(T) trajectories as an approximation since a CCSD­(T)-corrected dipole model is unavailable. Thus, its sampled nuclear configurations are determined by the CCSD­(T)-corrected potential-energy surface, whereas their dipole moments retain the DFT-revPBE-D3­(BJ) level description. All results are shown in Figure 6. We find all three models overestimate the static dielectric constant relative to experiment. That being said, ECW-TL-CCSD­(T) correctly reproduces its monotonic decrease with increasing temperature and exhibits a smooth temperature dependence, whereas the two DFT-based models display large fluctuations. Because the dielectric constant is governed by slowly converging collective dipole fluctuations, the overstructured liquids predicted by the DFT-based models may exhibit slower polarization relaxation, making their dielectric constants more difficult to converge. The remaining quantitative discrepancy for ECW-TL-CCSD­(T) probably arises from the approximate treatment of electronic polarization. Developing a consistent ECW-level dipole model trained on CW polarization data could further improve the predicted dielectric response and represents an important direction for future work.

**Figure 6:**
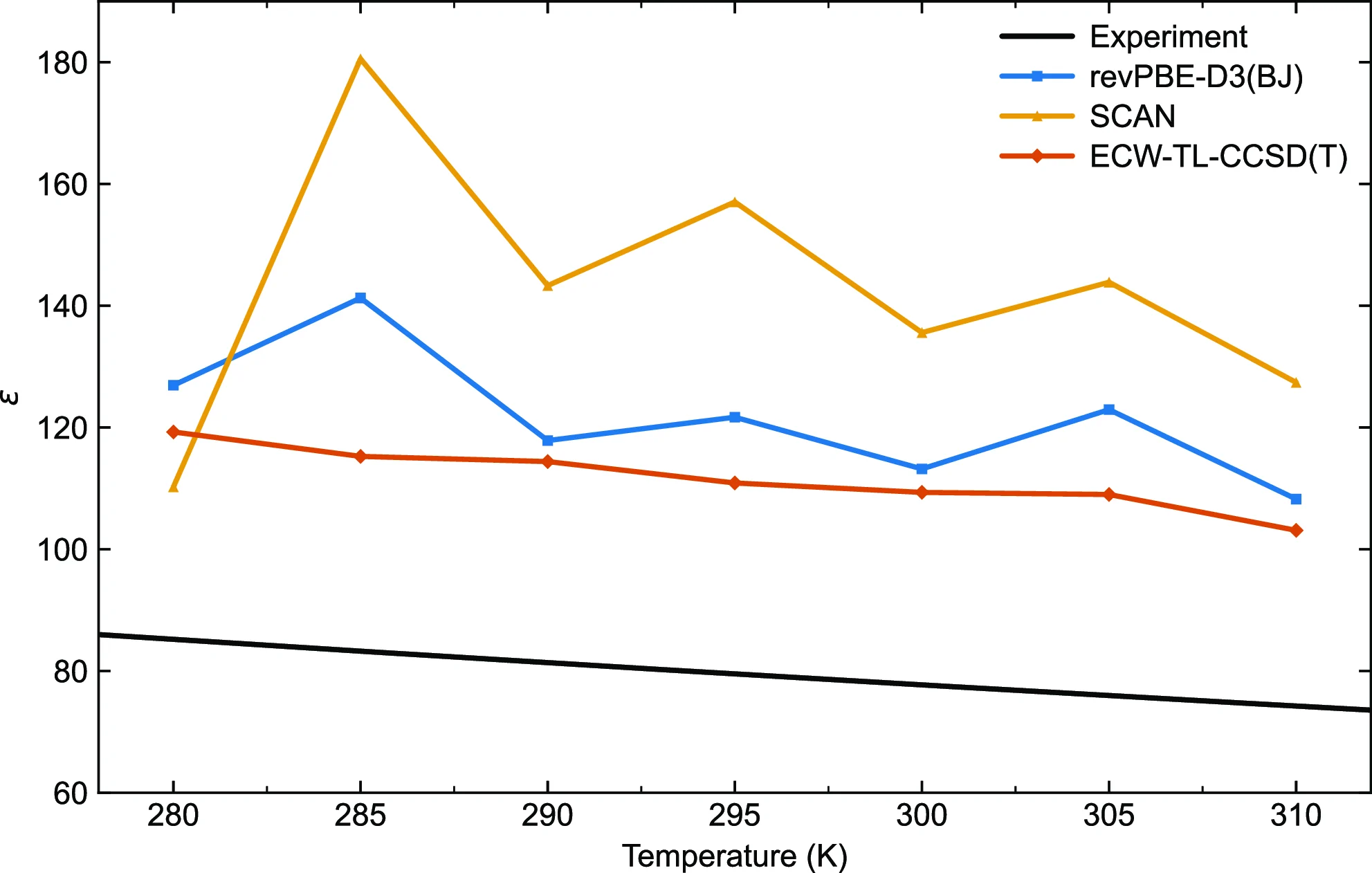
Temperature-dependent static dielectric constant *ε* of liquid water calculated from classical NPT-MD simulations using the three models, compared with experimental data from ref 111. Dipoles were evaluated using the revPBE-D3­(BJ) dipole model for both the revPBE-D3­(BJ) and ECW-TL-CCSD­(T) trajectories, and the SCAN dipole model for the SCAN trajectories. Although all three models overestimate the experimental values, ECW-TL-CCSD­(T) more consistently reproduces the temperature dependence and exhibits a smooth trend across the temperature range.

Finally, we calculate the infrared (IR) absorption spectrum from the time-correlation function of the total dipole moment. Classical MD simulations are performed for the cubic cell containing 512 water molecules at the experimental density. Each system is first equilibrated for 5 ps in the NVT ensemble at 298 K and then propagated in the microcanonical (NVE) ensemble with a time step of 0.25 fs to generate a production 50 ps trajectory. The instantaneous dipole moments are evaluated using the DW model corresponding to each DFT-based model. For the ECW-TL-CCSD­(T) trajectory, the DFT-revPBE-D3­(BJ) DW model is again used as an approximation. Within classical linear-response theory, the frequency-dependent absorption coefficient is calculated from the total-dipole autocorrelation function as

12 $$n\left(\omega \right)\alpha \left(\omega \right)=\frac{\omega^{2}}{6\varepsilon_{0}Vk_{B}Tc}\int_{-\infty }^{+\infty }e^{-i\omega t}\delta M\left(0\right)\cdot \delta M\left(t\right)\;dt$$

where δ ***M*** (*t*) = ***M*** (*t*) – ⟨ ***M*** ⟩. The results are shown in Figure 7.

**Figure 7:**
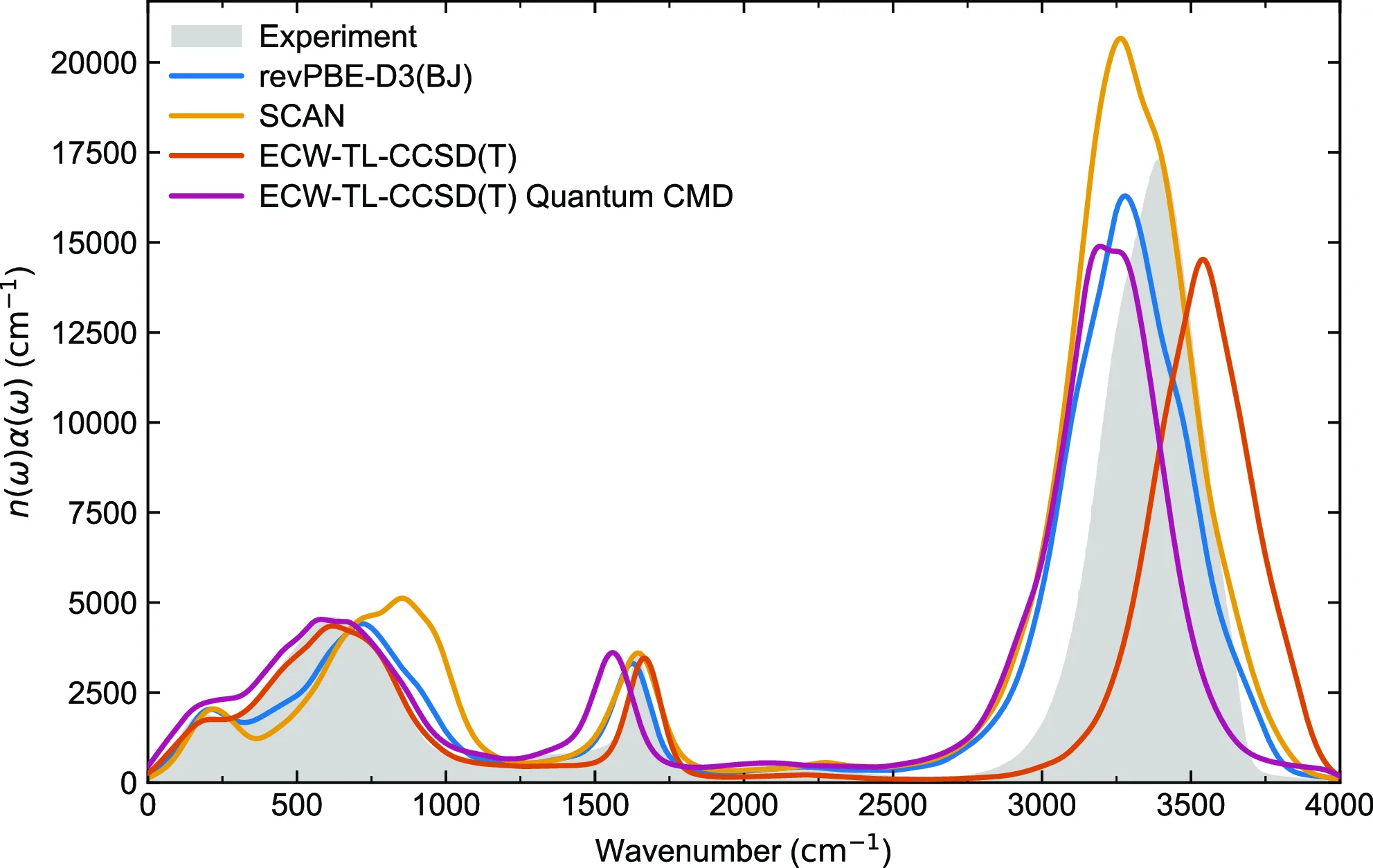
IR absorption spectra of liquid water calculated at 298 K and the experimental density. Classical NVE-MD results are shown for all three models, whereas quantum centroid MD results are shown only for ECW-TL-CCSD­(T). As in Figure 6, the SCAN dipole model was used for the SCAN spectra and the revPBE-D3­(BJ) dipole model for all other spectra. Experimental data are from ref 112. ECW-TL-CCSD­(T) improves the low-frequency intermolecular spectral features relative to both DFT-based models. Its classical O–H stretching band is blue-shifted relative to experiment, while its quantum CMD O–H stretching band is red-shifted, with the two results approximately bracketing the experimental peak.

In the low-frequency intermolecular region, ECW-TL-CCSD­(T) noticeably improves the peak positions and spectral line shape relative to both DFT-based models. In the intramolecular region, however, the classical ECW-TL-CCSD(T) spectrum overestimates the O–H stretching frequency, whereas both DFT-based models predict red-shifted stretching bands. To explore nuclear quantum effects, we additionally perform quantum centroid molecular dynamics^113^ (CMD) simulations for a smaller cell containing 64 water molecules using 32 ring-polymer beads and an adiabatic mass-separation parameter of 0.02.^114^ The improved description of the low-frequency intermolecular region is retained in CMD, while the O–H stretching band shifts to lower frequencies, consistent with the expected quantum red shift associated with nuclear zero-point motion on the anharmonic O–H stretching potential. However, quantum CMD is also known to produce an additional spurious red shift and broadening of high-frequency stretching bands because of the curvature problem in which fluctuations around the centroid artificially soften the effective O–H stretching potential of mean force.^115^ Therefore, the classical MD and quantum CMD spectra happen to bracket the experimental stretching frequency in the present calculations as approximate upper and lower bounds. For the DFT-based models, whose classical stretching bands are already red-shifted, nuclear quantum effects would likely shift them further away from experiment. Future calculations using improved approximate quantum dynamics methods can potentially provide a more reliable description of the O–H stretching band.^116,117^ Finally, because the ECW-TL-CCSD­(T) dipole moments are evaluated using the DFT-revPBE-D3­(BJ) DW model, this inconsistency probably introduces additional errors in the predicted spectral intensities and line shapes.

In this work, we extended the ECW-TL framework from systems characterized by spatially localized chemistry to homogeneous and dynamically disordered liquid phases through a multi-subsystem divide-and-conquer strategy. Using liquid water as a test case, we first demonstrated through controlled DFT-to-DFT validation that corrections evaluated for small, embedded subsystems can be assembled into full-system energy labels and used to improve a MLIP to a higher electronic-structure level. Applying the same procedure with embedded CCSD­(T) corrections yields the ECW-TL-CCSD­(T) potential, which provides consistent improvements across the structural, dynamical, thermodynamic, and dielectric properties examined here.

Despite these improvements, the ECW-TL-CCSD­(T) results do not yet agree quantitatively with experiments for all properties. The remaining discrepancies may arise from several sources. The first is the underlying embedding approximation. Although interactions within each subsystem are treated at the correlated-wavefunction level, correlations involving molecules assigned to different subsystems remain described at the baseline DFT level. One possible improvement would be to incorporate CW information self-consistently into the embedding potential through methods like potential functional embedding theory.^118–120^ Developing a computationally efficient implementation of such a self-consistent correlated embedding scheme is an interesting direction for future work. A second source of error is the transfer-learning procedure itself. The DFT-to-DFT validation provides a controlled and computationally accessible test of the multi-subsystem assembly and fine-tuning strategy, but it does not guarantee that the complete embedded CCSD­(T) correction has been learned with the available data. Residual errors may reflect incomplete sampling of local environments or limitations of the MLIP architecture in representing the correction. Systematic convergence tests with respect to the number and diversity of CW labels, together with more expressive machine-learning architectures could help distinguish data limitations from model limitations. A third source of uncertainty is the electronic-structure reference itself. Although CCSD­(T) is widely regarded as a high-accuracy method for molecular interactions, a definitive condensed-phase CCSD­(T) benchmark for liquid water remains difficult to establish. Developing and using systematically controlled low-scaling approximations to CCSD­(T), or other accurate and computationally tractable CW methods, would provide valuable independent benchmarks for assessing both the ECW approximation and the resulting transfer-learned potential.

The present demonstration is also limited to a chemically homogeneous liquid, for which partitioning into similarly sized local subsystems is relatively straightforward. Accurate many-body potentials such as MB-pol already provide excellent descriptions of pure water;^121^ the broader objective of ECW-TL is therefore to establish a transferable strategy for chemically diverse systems where similarly specialized potentials are unavailable. In solutions, the optimal partitioning may depend on the identities and strengths of the relevant interactions. A chemically informed scheme should retain the most important solute–solvent and reactive interactions within individual subsystems while assigning more weakly coupled and easy-to-describe interactions to the embedded environment. How such partitions should be selected, balanced, and adapted as the local chemical environment evolves remains to be systematically investigated.^122^

Extending the framework to solid–liquid, especially metal-solution interfaces presents additional challenges. At catalytic metal–electrolyte interfaces, bond breaking, charge transfer, and electronically near-degenerate configurations require multireference CW treatments.^51–56^ Applying ECW-TL to these systems will therefore require advances in both the transfer learning formalism and the electronic-structure method, where developing these capabilities is the focus of ongoing work. Moreover, the current MLIPs are short-ranged. Realistic simulations of heterogeneous and interfacial systems will require their combination with explicit long-range electrostatics,^123^ which further requires a CW-level dipole description consistent with the modern theory of polarization.^124^

Nevertheless, the consistent improvements in the structure, diffusion, density, and dielectric behavior of liquid water demonstrate the promise of divide-and-conquer ECW-TL for constructing correlated-wavefunction-quality condensed-phase interatomic potentials. Looking forward, the framework offers a scalable pathway toward chemically accurate simulations of complex liquids, solutions, and interfaces whose predictive description requires electronic-structure accuracy beyond standard DFT.

## Supplementary Material



## Data Availability

The training data, MLIP models, and input scripts to reproduce the simulations are available at 10.34770/q5q7-rq15.

## Notes

Computational details of the DFT calculations; DFET and ECW calculations; MLIP training, active learning and transfer-learning procedures; benchmarks of training batch-size and subsystem-size effects; validation of the reduced transfer-learning data set; and details of the MLWF calculations and DW model training (PDF)
