# Supplementary material for: A Divide-and-Conquer Embedded Correlated Wavefunction Transfer Learning Approach for Chemically Accurate Condensed-Phase Interatomic Potentials: Liquid Water as a Test Case
Source: J Phys Chem Lett. 2026 Sep 29;17(40):11430–42. doi: 10.1021/acs.jpclett.6c02897 (PMC13656776; doi:10.1021/acs.jpclett.6c02897)
Supplement: Supplementary file 1 [file jz6c02897_si_001.pdf]

Supporting Information: A Divide-and-Conquer Embedded Correlated Wavefunction Transfer Learning Approach for Chemically Accurate Condensed-Phase Interatomic Potentials: Liquid Water as a Test Case

Xuezhi Bian[1, †] and Emily A. Carter[2,3, *]

[1]Department of Chemistry, Princeton University, Princeton, New Jersey 08544, United States

[2]Department of Mechanical and Aerospace Engineering, Princeton University, Princeton, New Jersey 08544, United States

[3]Andlinger Center for Energy and the Environment and Program in Applied and Computational Mathematics, Princeton University, Princeton, New Jersey 08544, United States

Corresponding email address: *eac@princeton.edu; †xzbian@princeton.edu

Computational details of the density functional theory calculations

All periodic plane-wave density functional theory (DFT) calculations used to train the DFT-based machine-learned interatomic potentials (MLIPs) were performed using VASP[1,2] within the all-electron frozen-core projector augmented-wave (PAW) formalism.[3,4] The standard PBE PAW potentials were used for both O and H, with a plane-wave kinetic-energy cutoff of 600 eV. Brillouin-zone sampling was restricted to the Γ point. Gaussian smearing with a width of 0.1 eV was employed for electronic convergence, and the self-consistent-field (SCF) energy convergence threshold was set to 10^{-6} eV.

Computational details of density functional embedding theory and embedded correlated wavefunction theory calculations

For the embedding potential optimization in density functional embedding theory (DFET), a real-space grid of size 216 × 216 × 216 was used.[5] The PAW derivative-correction scaling factor parameter F_{corr} was set to 0.70, which restricts the correction to points satisfying $r \leq F_{\mathrm{corr}} R_{\mathrm{aug}}$, where R_{aug} is the PAW augmentation radius.[6] This truncation reduces computational cost by neglecting the small correction near the boundary of the augmentation sphere. The embedding potential was optimized by maximizing the multi-subsystem extended Wu-Yang functional (Eq. 5 in the main text) until the gradient norm was below 10^{-5}. The optimized embedding potential then was projected onto a Gaussian-type-orbital (GTO) basis using the EmbeddingIntegralGenerator code for the ECW calculations.[7]

All embedded canonical CCSD(T) and GTO-DFT calculations were performed using PySCF[8] with the cc-pVTZ basis set.[9] The CCSD(T) calculations employed converged Hartree-Fock (HF) reference wavefunctions. Density fitting was used to accelerate the DFT and HF GTO calculations with the cc-pVTZ-jkfit auxiliary basis,[10] while the cc-pVTZ-RI auxiliary basis[11] was employed for

the CCSD(T) calculations. The GTO-DFT and GTO-HF SCF calculations were converged to an energy threshold of 10^{-9} Hartree, and the CCSD(T) calculations were converged to 10^{-7} Hartree.

Details of MLIP training, active learning and transfer learning

All MLIP models employed the Deep Potential (DP) model architecture, as implemented in DeePMD-kit,[12] using the standard short-range, smooth-edition, two-body descriptor with a cutoff radius of $r_c = 6$ Å. The embedding network contains three hidden layers containing 25, 50, and 100 neurons, respectively, with 12 axis neurons. The fitting network contains three hidden layers of 240 neurons each. Training was performed for 400,000 steps using a batch size of one configuration. The learning rate was scheduled to decrease exponentially throughout training from 10^{-3} to 10^{-8}. The energy and virial prefactors in the loss function were increased from 0.02 to 1, whereas the force prefactor was decreased from 1000 to 1. These network architectures and training parameters were selected based on previous DP studies of liquid water.[13]

Configuration space was explored using the query-by-committee active-learning workflow implemented in DP-GEN.[14] Each active-learning cycle contains three stages: training, exploration, and labeling. During training, four MLIP models were initialized with different random seeds and trained on the same dataset. During exploration, these models were used to perform molecular dynamics (MD) simulations under the thermodynamic conditions relevant to the target applications. The uncertainty of each sampled configuration was quantified by the force model deviation among the four models. Configurations with deviations between 0.15 and 0.35 eV/Å were identified as candidates for labeling. In the labeling stage, DFT calculations were performed following the procedure above to generate reference energies, forces, and virials, which were then added to the training dataset for the next iteration. Separate active-learning workflows were performed for the DFT-revPBE-D3(BJ) and DFT-SCAN MLIPs, each starting from 100 configurations selected from DFT-MD trajectories. The initial exploration stage employed classical NPT simulations over the temperature range of 250-350 K at pressure of 1 bar. Convergence was reached after 16 active-learning iterations for DFT-revPBE-D3(BJ) and 13 iterations for DFT-SCAN, with more than 99% of the sampled configurations exhibiting force-model deviations below 0.15 eV/Å. The resulting datasets contained 666 and 522 configurations, respectively, and the corresponding models were used for the validation tests reported in the main text.

The exploration space was then extended by performing path-integral molecular dynamics (PIMD) simulations with LAMMPS[15,16] over the same temperature range and pressure conditions to incorporate configurations arising from nuclear quantum fluctuations. The number of beads, time step, and thermostat and barostat parameters in these PIMD simulations were identical to those specified for the production PIMD simulations in the main text. Applying the same convergence criterion, the DFT-revPBE-D3(BJ) and DFT-SCAN workflows converged after 34 and 32 active-learning iterations, respectively. The final datasets contained 2,379 DFT-revPBE-D3(BJ)

configurations and 2,222 DFT-SCAN configurations. The resulting models were used as the baseline models for transfer learning and for the MD simulations reported in the application section.

All transfer learning was initialized from the baseline DFT-revPBE-D3(BJ) model, with the network architecture kept unchanged. Because the ECW calculations provided only energy corrections, the energy prefactor in the loss function was fixed at 1, whereas the force and virial prefactors were set to 0. The learning rate was decayed exponentially from 10^{-6} to 10^{-8}. This smaller starting learning rate was used to limit catastrophic forgetting of the baseline potential during fine tuning.

Benchmarks of batch-size effects

As discussed in the main text, we found the batch size is the most influential hyperparameter in the fine-tuning procedure. Here we repeated the transfer learning using batch sizes of 1, 10, and 100. The corresponding numbers of training steps were 1,000,000, 100,000 and 10,000, respectively, such that the total number of configurations processed during training remained constant. All other settings, including the training dataset and MD simulation details, were identical to those used for Fig. 2 of the main text. As shown in Fig. S1, a batch size of 100 produces the best agreement of $g_{OO}(r)$ with the DFT-SCAN reference. We therefore used a batch size of 100 for all fine-tuning calculations reported in the main text, with each model fine-tuned for 10,000 steps.

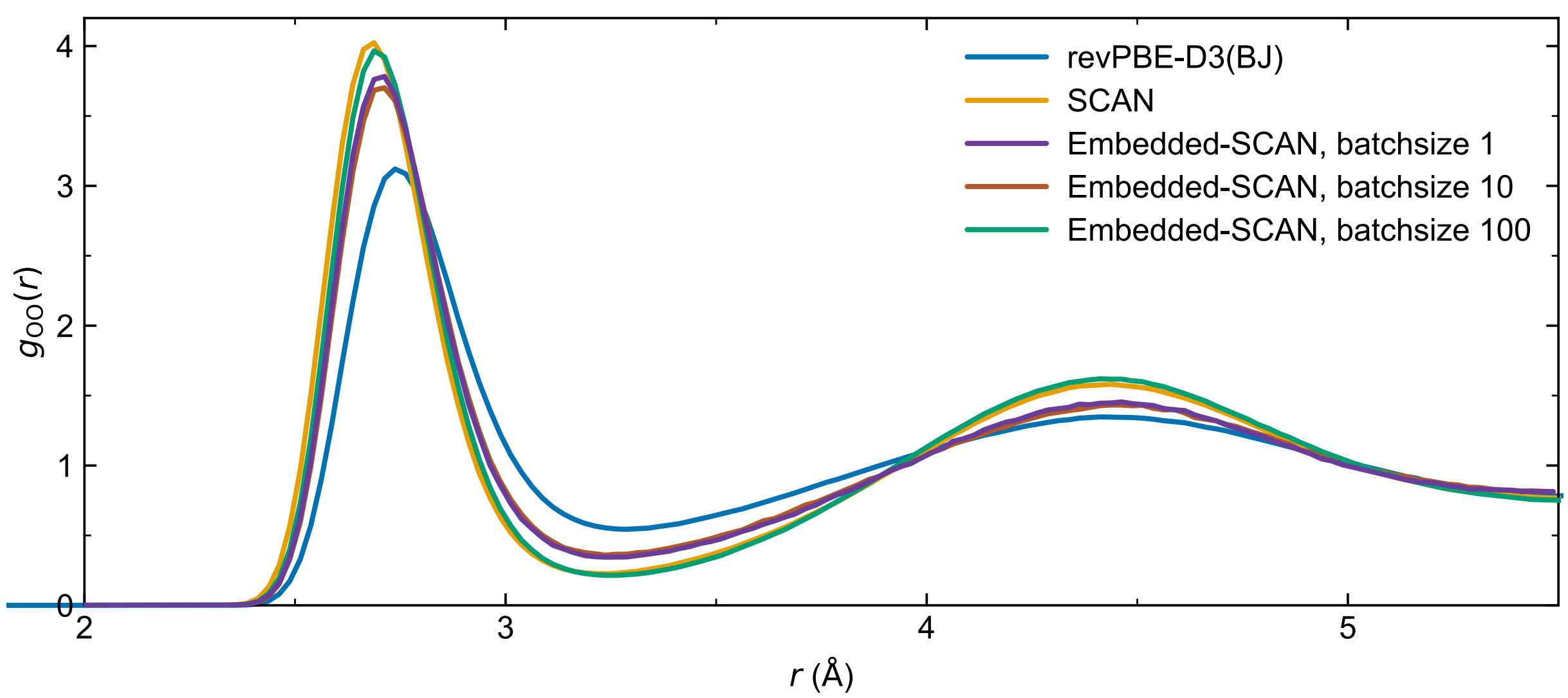

Figure S1 Oxygen-oxygen radial distribution (RDFs) functions $g_{OO}(r)$ of liquid water obtained from classical NVT-MD simulations at 298 K and the experimental density using DFT-revPBE-D3(BJ), DFT-SCAN, and embedded-SCAN models fine-tuned with different batch sizes. The largest batch size yields the closest agreement with the DFT-SCAN reference.

Benchmarks of subsystem-size effects

To test how the performance of divide-and-conquer DFET depends on the number and size of the subsystems, we used the 666-configuration DFT-revPBE-D3(BJ) validation dataset and partitioned each 64-water system into 2, 4, or 16 subsystems containing 32, 16, or 4 water molecules, respectively, in addition to the 8-subsystem case presented in the main text. Separate DFET calculations were performed for each partitioning scheme, followed by embedded DFT-SCAN calculations. The resulting subsystem energy corrections were assembled into full-system corrections and used for transfer learning. Classical NVT-MD simulations then were performed using the same settings as in Fig. 2 of the main text. As shown in Fig. S2, all models fine-tuned using different numbers of subsystems agree quite well with the DFT-SCAN reference. The results obtained using 2, 4, and 8 subsystems nearly overlap, whereas the 16-subsystem model shows a small reduction in accuracy. We therefore selected eight subsystems as the best compromise between accuracy and computational cost throughout this work.

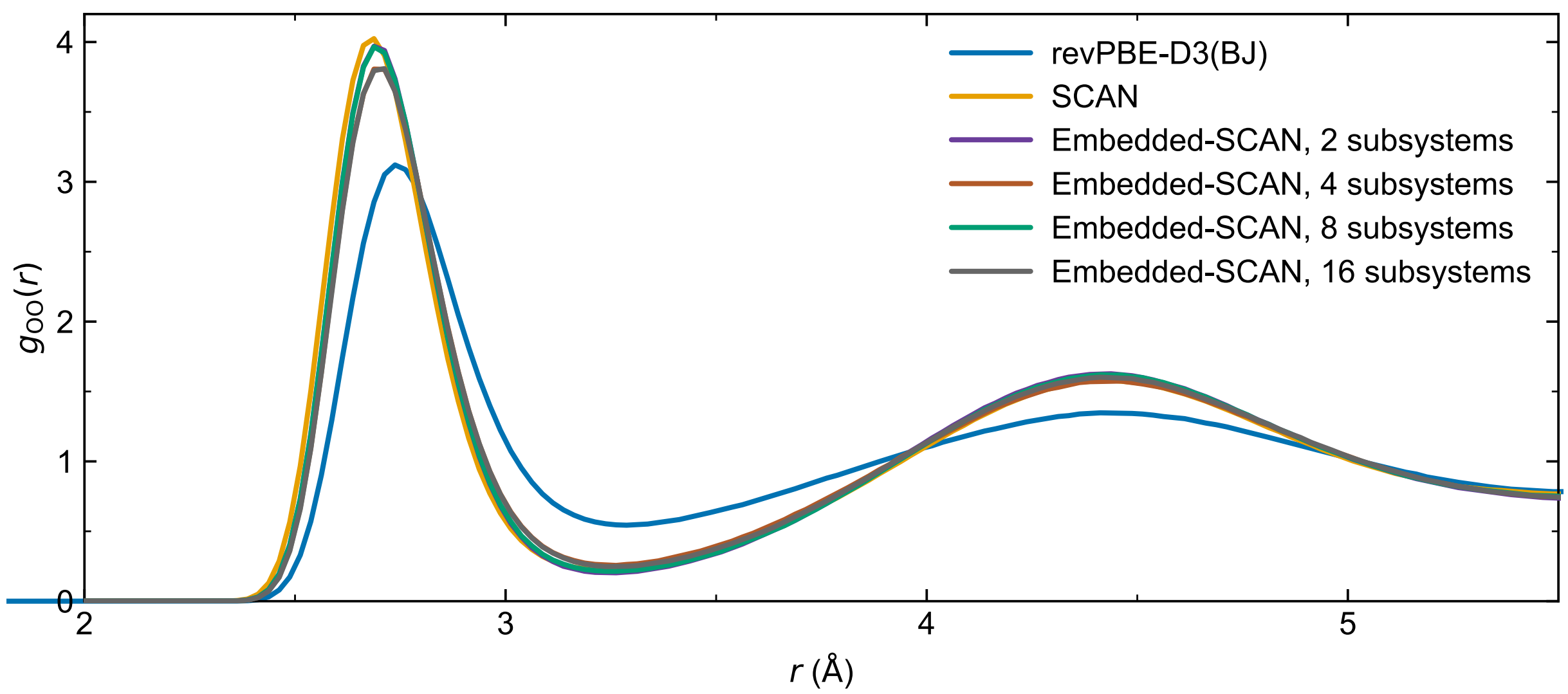

Figure S2 Oxygen-oxygen RDFs $g_{OO}(r)$ of liquid water obtained from classical NVT-MD simulations at 298 K and the experimental density using DFT-revPBE-D3(BJ), DFT-SCAN, and embedded-SCAN models fine-tuned with corrections computed with different subsystem sizes. The results obtained with less than 8 subsystems are nearly identical.

Validation of the reduced transfer-learning dataset

To reduce the computational cost of the ECW calculations, we selected a smaller dataset of 300 configurations from the baseline DFT-revPBE-D3(BJ) training dataset and from MD trajectories generated at 300 and 350 K using the baseline model. Configurations with large force deviations, as predicted by the committee of four independently trained models from the final active-learning iteration of the baseline model, were selected. To validate this reduced dataset, the baseline model was fine-tuned using embedded DFT-SCAN energy corrections for the selected configurations. An additional 100 configurations were selected using the same procedure and reserved for validation. Figure S3 compares the embedded-SCAN finetuned model predicted energies and forces with the

reference embedded-SCAN energies and full-cell SCAN forces, respectively. Full-cell SCAN forces were used because embedded-SCAN forces were unavailable. The fine-tuned model exhibits small and comparable energy and force RMSEs on the training and validation datasets, supporting the suitability of the 300-configuration dataset for fine-tuning to embedded SCAN.

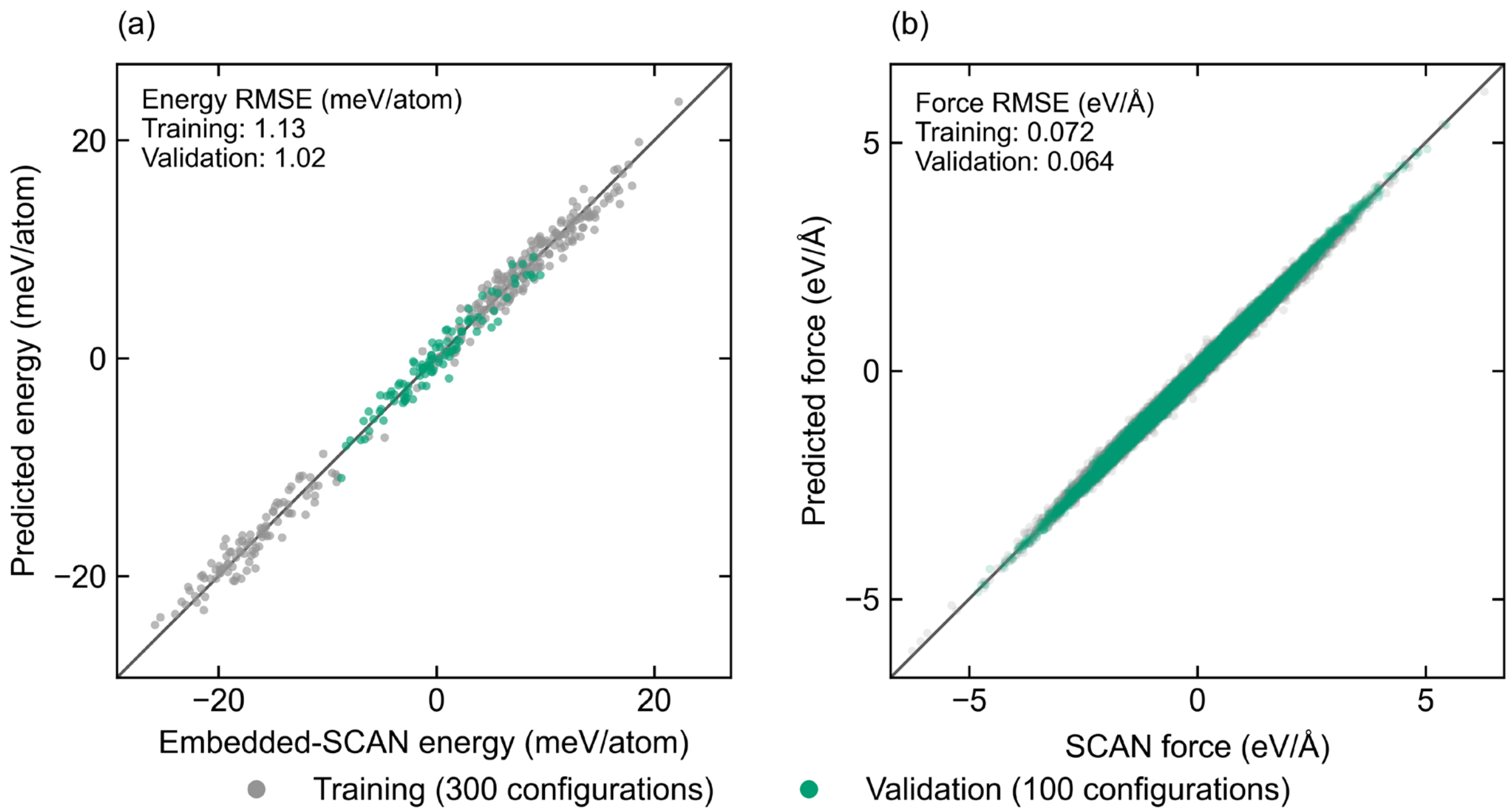

Figure S3 Energy and force parity plots for the embedded-SCAN fine-tuned model on the training (300 configurations) and validation (100 configurations) datasets. Predicted energies and forces are compared with reference embedded-SCAN energies and full-cell SCAN forces, respectively. The model exhibits comparable RMSEs on the training and validation datasets.

To further validate the model, NVT-MD simulations were performed using the same settings as in Fig. 2 of the main text. As shown in Fig. S4, transfer learning with only 300 energy corrections closely reproduces the DFT-SCAN reference RDF. These results provide further support for using this reduced dataset in subsequent fine-tuning with ECW corrections.

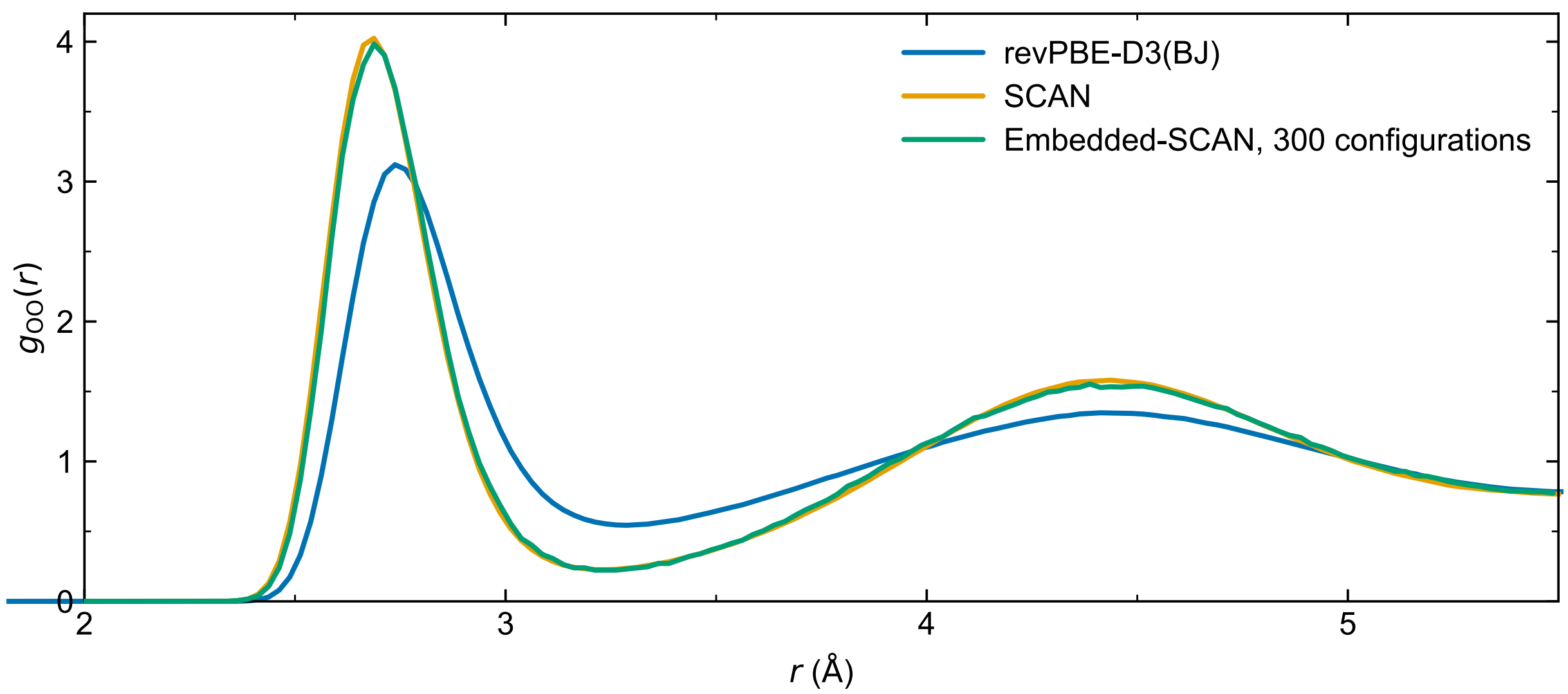

Figure S4 Oxygen-oxygen RDFs $g_{OO}(r)$ of liquid water obtained from classical NVT-MD simulations at 298 K and the experimental density using DFT-revPBE-D3(BJ), DFT-SCAN, and embedded-SCAN. The embedded-SCAN model was fine-tuned using corrections calculated for a reduced dataset of 300 configurations and closely reproduces the reference DFT-SCAN RDF.

Computational details of maximally localized Wannier function calculations and Deep Wannier model training

To study the dielectric properties of liquid water, maximally localized Wannier functions (MLWFs) were computed using Wannier90[17] for the complete DFT training datasets of both the DFT-revPBE-D3(BJ) and DFT-SCAN models. For each simulation cell containing 64 water molecules, 256 MLWFs were constructed from the 256 fully occupied valence bands. The Wannierization process was converged to a threshold of 10^{-8}. The four Wannier centers closest to each oxygen atom could be assigned unambiguously as representing its eight valence electrons. Their mean displacement relative to the atom was used as the Wannier-centroid training label.

A separate Deep Wannier (DW) model was trained for each DFT functional to predict the Wannier-centroid displacement associated with each oxygen atom. The DW models employed the same three-layer embedding and fitting network architectures as the corresponding energy-and-force models, together with the same exponentially decaying learning-rate schedule. Each model was trained for 1,000,000 steps. The resulting DW models were used to evaluate the dielectric properties reported in the main text.

References

(1) Kresse, G.; Hafner, J. Ab Initio Molecular Dynamics for Liquid Metals. *Phys. Rev. B* **1993**, *47* (1), 558–561. https://doi.org/10.1103/PhysRevB.47.558.

(2) Kresse, G.; Furthmüller, J. Efficient Iterative Schemes for Ab Initio Total-Energy Calculations Using a Plane-Wave Basis Set. *Phys. Rev. B* **1996**, *54* (16), 11169–11186. https://doi.org/10.1103/PhysRevB.54.11169.
(3) Blöchl, P. E. Projector Augmented-Wave Method. *Phys. Rev. B* **1994**, *50* (24), 17953–17979. https://doi.org/10.1103/PhysRevB.50.17953.
(4) Kresse, G.; Joubert, D. From Ultrasoft Pseudopotentials to the Projector Augmented-Wave Method. *Phys. Rev. B* **1999**, *59* (3), 1758–1775. https://doi.org/10.1103/PhysRevB.59.1758.
(5) Yu, K.; Carter, E. A. *VASPEmbedding*. https://github.com/EACcodes/VASPEmbedding.
(6) Yu, K.; Libisch, F.; Carter, E. A. Implementation of Density Functional Embedding Theory within the Projector-Augmented-Wave Method and Applications to Semiconductor Defect States. *J. Chem. Phys.* **2015**, *143* (10), 102806. https://doi.org/10.1063/1.4922260.
(7) Krauter, C. M.; Carter, E. A. EmbeddingIntegralGenerator. https://github.com/EACcodes/EmbeddingIntegralGenerator.
(8) Sun, Q.; Zhang, X.; Banerjee, S.; Bao, P.; Barbry, M.; Blunt, N. S.; Bogdanov, N. A.; Booth, G. H.; Chen, J.; Cui, Z.-H.; Eriksen, J. J.; Gao, Y.; Guo, S.; Hermann, J.; Hermes, M. R.; Koh, K.; Koval, P.; Lehtola, S.; Li, Z.; Liu, J.; Mardirossian, N.; McClain, J. D.; Motta, M.; Mussard, B.; Pham, H. Q.; Pulkin, A.; Purwanto, W.; Robinson, P. J.; Ronca, E.; Sayfutyarova, E. R.; Scheurer, M.; Schurkus, H. F.; Smith, J. E. T.; Sun, C.; Sun, S.-N.; Upadhyay, S.; Wagner, L. K.; Wang, X.; White, A.; Whitfield, J. D.; Williamson, M. J.; Wouters, S.; Yang, J.; Yu, J. M.; Zhu, T.; Berkelbach, T. C.; Sharma, S.; Sokolov, A. Yu.; Chan, G. K.-L. Recent Developments in the PySCF Program Package. *J. Chem. Phys.* **2020**, *153* (2), 024109. https://doi.org/10.1063/5.0006074.
(9) Dunning, T. H. Gaussian Basis Sets for Use in Correlated Molecular Calculations. I. The Atoms Boron through Neon and Hydrogen. *J. Chem. Phys.* **1989**, *90* (2), 1007–1023. https://doi.org/10.1063/1.456153.
(10) Weigend, F. A Fully Direct RI-HF Algorithm: Implementation, Optimised Auxiliary Basis Sets, Demonstration of Accuracy and Efficiency. *Phys. Chem. Chem. Phys.* **2002**, *4* (18), 4285–4291. https://doi.org/10.1039/b204199p.
(11) Weigend, F.; Köhn, A.; Hättig, C. Efficient Use of the Correlation Consistent Basis Sets in Resolution of the Identity MP2 Calculations. *J. Chem. Phys.* **2002**, *116* (8), 3175–3183. https://doi.org/10.1063/1.1445115.
(12) Zeng, J.; Zhang, D.; Peng, A.; Zhang, X.; He, S.; Wang, Y.; Liu, X.; Bi, H.; Li, Y.; Cai, C.; Zhang, C.; Du, Y.; Zhu, J.-X.; Mo, P.; Huang, Z.; Zeng, Q.; Shi, S.; Qin, X.; Yu, Z.; Luo, C.; Ding, Y.; Liu, Y.-P.; Shi, R.; Wang, Z.; Bore, S. L.; Chang, J.; Deng, Z.; Ding, Z.; Han, S.; Jiang, W.; Ke, G.; Liu, Z.; Lu, D.; Muraoka, K.; Oliaei, H.; Singh, A. K.; Que, H.; Xu, W.; Xu, Z.; Zhuang, Y.-B.; Dai, J.; Giese, T. J.; Jia, W.; Xu, B.; York, D. M.; Zhang, L.; Wang, H. DeePMD-Kit v3: A Multiple-Backend Framework for Machine Learning Potentials. *J. Chem. Theory Comput.* **2025**, *21* (9), 4375–4385. https://doi.org/10.1021/acs.jctc.5c00340.
(13) Zhang, L.; Wang, H.; Car, R.; E, W. Phase Diagram of a Deep Potential Water Model. *Phys. Rev. Lett.* **2021**, *126* (23), 236001. https://doi.org/10.1103/PhysRevLett.126.236001.
(14) Zhang, Y.; Wang, H.; Chen, W.; Zeng, J.; Zhang, L.; Wang, H.; E, W. DP-GEN: A Concurrent Learning Platform for the Generation of Reliable Deep Learning Based Potential Energy Models. *Comput. Phys. Commun.* **2020**, *253*, 107206. https://doi.org/10.1016/j.cpc.2020.107206.
(15) Thompson, A. P.; Aktulga, H. M.; Berger, R.; Bolintineanu, D. S.; Brown, W. M.; Crozier, P. S.; in 't Veld, P. J.; Kohlmeyer, A.; Moore, S. G.; Nguyen, T. D.; Shan, R.; Stevens, M. J.;

Tranchida, J.; Trott, C.; Plimpton, S. J. LAMMPS - a Flexible Simulation Tool for Particle-Based Materials Modeling at the Atomic, Meso, and Continuum Scales. *Comput. Phys. Commun.* **2022**, *271*, 108171. https://doi.org/10.1016/j.cpc.2021.108171.

(16) Li, Y.; Gomez, A.; Cai, K.; Zhang, C.; Fu, L.; Jia, W.; Feldman, Y. M. Y.; Blumer, O.; Higer, J.; Hirshberg, B.; Xu, S.; Kohlmeyer, A.; Car, R. Fix Pimd/Langevin: An Efficient Implementation of Path Integral Molecular Dynamics in LAMMPS. *J. Chem. Phys.* **2026**, *164* (14), 144119. https://doi.org/10.1063/5.0328429.

(17) Pizzi, G.; Vitale, V.; Arita, R.; Blügel, S.; Freimuth, F.; Géranton, G.; Gibertini, M.; Gresch, D.; Johnson, C.; Koretsune, T.; Ibañez-Azpiroz, J.; Lee, H.; Lihm, J.-M.; Marchand, D.; Marrazzo, A.; Mokrousov, Y.; Mustafa, J. I.; Nohara, Y.; Nomura, Y.; Paulatto, L.; Poncé, S.; Ponweiser, T.; Qiao, J.; Thöle, F.; Tsirkin, S. S.; Wierzbowska, M.; Marzari, N.; Vanderbilt, D.; Souza, I.; Mostofi, A. A.; Yates, J. R. Wannier90 as a Community Code: New Features and Applications. *J. Phys. Condens. Matter* **2020**, *32* (16), 165902. https://doi.org/10.1088/1361-648X/ab51ff.
